# Risk of Cognitive Decline in Women with Parkinson’s Disease Is Reduced by Early Age at Menarche

**DOI:** 10.3390/neurolint17100161

**Published:** 2025-10-05

**Authors:** Giuseppe Schirò, Carlo Fazio, Paolo Aridon, Cesare Gagliardo, Chiara Davì, Valentina Picciolo, Tiziana Colletti, Chiara Tumminia, Salvatore Iacono, Paolo Ragonese, Marco D’Amelio

**Affiliations:** 1 Department of Biomedicine, Neurosciences and Advanced Diagnostics (BiND), University of Palermo, 90127 Palermo, Italy; carlo.fazio01@universitadipavia.it (C.F.); paolo.aridon@unipa.it (P.A.); cesare.gagliardo@unipa.it (C.G.); chiaradavi092@gmail.com (C.D.); valentinapicciolo.vp@gmail.com (V.P.); tizianacolletti@gmail.com (T.C.); chiara.tumminia@unipa.it (C.T.); salvo.iak@gmail.com (S.I.); paolo.ragonese@unipa.it (P.R.); 2 Neurology and Multiple Sclerosis Center, Unità Operativa Complessa (UOC), Foundation Institute “G. Giglio”, 90015 Cefalù, Italy

**Keywords:** menarche, menopause, neuroplasticity, cognitive decline

## Abstract

Background: Parkinson’s disease (PD) is a neurodegenerative disorder affecting men more frequently than women, a difference that might be due to many factors, including sexual hormones. Estrogens seem to confer a protective effect on the nigrostriatal pathway in experimental studies but their effects on cognition in patients with PD are unknown. Aim: To investigate the impact of the exogenous and endogenous estrogens on cognitive impairment in women with PD. Methods and materials: We recruited and consecutively interviewed outpatient women affected by PD. Each patient underwent a cognitive assessment via the Montreal Cognitive Assessment scale (MoCA), an anamnestic collection of the reproductive lifespan variables and clinical features. We investigated if some of the reproductive lifespan variables investigated could predict cognition outcomes in post-menopausal women with PD. Results: A total of 90 women with PD were recruited. Women with MoCA ≥ 26 (*n* = 27) had a lower median age at menarche (11 [11,12] vs. 13 [12–14], *p* < 0.0001), lower disease duration in years (8.3 [6.1–12.7] vs. 9.4 [6–12.7], *p* = 0.6), and less advanced disease (1 [1,2] vs. 2 [1–3], *p* = 0.02). Among all the reproductive life-span variables, only earlier age at menarche significantly predicted higher scores on MoCA (aOR = 0.5 [0.3–0.8], *p* = 0.005). No other clinical and reproductive factors have been shown to have an influence on cognitive scores. Conclusions: Age at menarche correlated with cognitive outcomes. Our study suggests that earlier exposure to endogenous estrogens during a phase of development and plasticity of the brain might preserve women with PD from cognitive decline.

## 1. Introduction

Parkinson’s disease (PD) is a neurodegenerative disease that affects both men and women with a greater risk for men, a difference that may possibly be due to a protective role of estrogens [1]. Numerous experimental investigations have suggested that estrogens may have neuroprotective functions on the dopaminergic system. It is known that pre-treatment with estrogens promotes the survival of dopaminergic neurons from death caused by 1-methyl-4-phenyl-1,2,3,6-tetrahydropyridine (MPTP)-induced neurotoxicity, an experimental model of PD [2]. Other results seem to suggest that estrogens are required for the plasticity phenomena of the nigro-striatal system [3], and they seem necessary to promote the differentiation, growth, and survival of neurons [3,4,5,6]. Several epidemiological studies on humans also confirm a possible protective role of estrogens on the central nervous system (CNS). A longer duration of fertile life and, therefore, a longer exposure to estrogens are supposed to delay the age of PD onset in women [1]. Moreover, age at menopause has been correlated with age of PD onset [7,8], suggesting that the use of exogenous estrogens, such as the administration of oral contraceptives, increases the risk of PD [9]. Little is known about the impact of estrogens on the cognitive decline of women with PD. A recent paper showed that in healthy midlife women, the density of estrogen receptors increases in the brain during the transition to menopause. Higher density of estrogen receptors in target regions, such as hippocampus, amygdala, and frontal cortex, were associated with lower performances in cognitive tasks [10]. It is unknown if this finding is or is not a compensatory mechanism, for instance, of the decline in estrogens levels during menopause or of the gray matter volume loss that is observed in menopausal women.

Based on this last consideration, we hypothesized a protective role of estrogens, both endogenous and exogenous, and conducted a cross-sectional study to (1) evaluate the influence of reproductive life factors on the cognitive outcomes of patients with PD and (2) consider other possible aspects that can be influenced by exposure to estrogens, such as motor and non-motor manifestations of PD, and the stage of the disease.

## 2. Methods and Materials

### 2.1. Ethical Statement

The study was conducted in accordance with the Declaration of Helsinki and approved by the Ethics Committee of “Policlinico Paolo Giaccone”, Palermo, Italy. Informed consent was obtained from each participant.

### 2.2. Study Design and Participants

Outpatients of the “Movement Disorders and Parkinson’s Disease” Center, at the “Paolo Giaccone” University Hospital of Palermo, were recruited during the period between December 2022 and December 2023. All patients included in the study were post-menopausal women who had an established diagnosis of PD, according to the Queen Square Bank criteria. Each patient underwent a cognitive assessment using the Montreal Cognitive Assessment scale (MoCA). We used MoCA scores adjusted for age and education. No specific MoCA sub-scores were analyzed. Scores less than 26 were chosen to define the presence of cognitive decline (CD) [11].

### 2.3. Clinical Features Evaluated

Demographic and clinical data of the patients (age of onset, disease duration, age at evaluation) were collected. Stage of disease was classified according to the Hoen and Yahr (H&Y) classification. The equivalent dose of levodopa (LEDD) was collected for each patient. LEDD were calculated by converting total daily antiparkinsonian medications according to the currently used criteria.

### 2.4. Reproductive Lifespan Variables Investigated

Fertile life data collected regarded (1) age at menarche; (2) age at menopause; (3) type of menopause (spontaneous vs. surgical); (4) the administration of estrogen–progestin (EP) contraceptives during fertile life; (5) the use of hormone replacement therapy (HRT) following menopause; (6) the history of delivery and the number of deliveries; (7) the history of pregnancy, the numbers of pregnancies and cumulative duration of pregnancy in months and indicated as cumulative length of pregnancy (CLP); (8) eventual breastfeeding (including total time dedicated to breastfeeding calculated in months); (9) history of abortion and numbers of abortions; (10) fertile life length was calculated as the difference between age at menarche and age at menopause, calculated in months. Data on fertile life were retrospectively acquired by examining the medical records or by requiring medical documentation for the patients who never self-reported.

### 2.5. Statistical Analyses

The Shapiro–Wilks test was applied to verify the distribution of the variables. Parametric continuous variables were reported as means and standard deviations, while skewed data were reported as medians with interquartile ranges (IQR). Unconditional univariate and multivariate logistic regression analysis were conducted to evaluate the predictors of MoCA. We considered pathological value scores of MoCA lower than 26. The clinical and fertile life factors were used as independent variables. Age at menarche, age at menopause, duration of fertility, total time of breastfeeding, LGC, number of abortions, number of deliveries, HRP duration, EP duration, age at HRP starting, age at EP starting were evaluated as continuous variables. Types of menopause (natural versus surgical), history of abortion, history of pregnancy, and breast feeding were analyzed as categorical variables. We calculated the odds ratios (OR) with 95% confidence intervals (CI) and two tailed *p* values (alpha = 0.05) by using univariate and multivariate regression models. Due to the limited sample size with many reproductive lifespan variables and to avoid the risk of overfitting, we limited the number of predictors included in the multivariate analysis considering only the variables resulted significantly associated at the univariate analysis. The statistical analyses were performed using SPSS (IBM Corp. Released 2019. IBM SPSS Statistics for MacOS, Version 26.0. Armonk, NY, USA: IBM Corp.). For all analyses carried out, the level of statistical significance was set less than or equal to 0.05.

## 3. Results

Ninety Caucasian female patients with PD were recruited during the study period. All the demographic and clinical features are shown in Table 1. Table 2 reports differences between patients with PD according to pathological or normal MoCA scores. Categorical variables were analyzed by chi-square test, while continuous variables were calculated by Mann–Whitney test, as appropriate. All variables have shown a non-parametric distribution.

### Clinical Variables and Factors of Fertile Life Associated with MoCA

MoCA was considered pathological when a patient scored less than 26. As shown in Table 3, among the clinical predictors at univariate analysis, age at evaluation, HY and UPDRS values were related to scores on MoCA. Among the fertile life predictors, the univariate logistic regression showed a significant association of age at menarche and EP therapy with MoCA scores. However, at multivariate logistic regression, we found that only the age at menarche (aOR = 0.5 [0.3–0.8], *p* = 0.005) was the strongest predictor of MoCA scores. In particular, younger age at menarche is associated with higher performances on MoCA.

**Figure 1:**
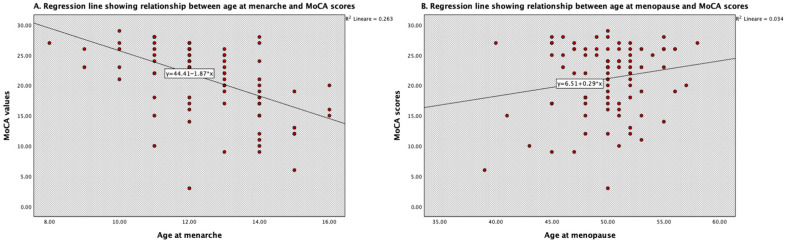
Scatter plots for relationship between (**A**) cognitive scores and age at menarche and between (**B**) cognitive scores and age at menopause.The asterisk (*) indicates statistical significance (*p* < 0.05).

Figure 1 describes the linear relationship between cognitive score and, respectively, age at menarche and age at menopause. In particular, scores of cognitive performance significantly decrease as age at menarche increases and as age at menopause decreases. However, only age at menarche reached the statistical significance (*p* = 0.005). As indicated by the value of R^2^ = 0.263, the 26.3% variability of MoCA scores may be attributed to age at menarche. On the other hand, R^2^ = 0.034 indicated that only 3.4% of MoCA scores may be influenced by age at menopause. Consequently, a clearer association with age at menarche can be outlined.

**Figure 2:**
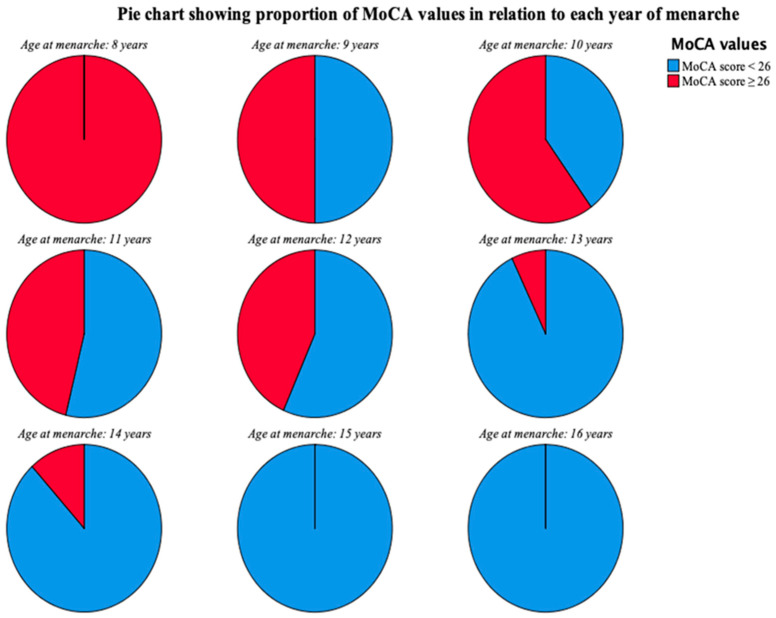
Repartition on pie chart of MoCA score ***≥*** 26 in relation to each year of menarche observed in our cohort. MoCA score *≥* 26 was 100% at 8 years, 50% at 9 years, 60% at 10 years, 46.2% at 11 years, 43.3% at 12 years, 7.1% at 13% years, 11.8% at 14 years, and 0% at 15 and 16 years. Dramatic reduction in MoCA scores ***≥*** 26 was seen in women having menarche after 12 years of age.

In the following pie chart (Figure 2), the large concentration of patients with MoCA scores compatible with cognitive decline is distributed in the group with the latest years of menarche. In particular, after 12 years of age, there is a clear decline from 43.3% to 7.1% of people with MoCA ≥ 26, suggesting that menarche by 12 years of age provides protection against the subsequent development of cognitive decline.

## 4. Discussion

This cross-sectional study explored the association of the reproductive lifespan variables with cognitive and motor outcomes in a cohort of women with PD.

### 4.1. Menarche, Menopause, and Duration of Fertility

We reported a direct correlation between age at menarche and MoCA scores, since people with PD and an early menarche scored better on the cognitive assessment. Our findings suggest that a late exposure to the endogenous estrogens can affect cognition in women with PD. Estrogens exert a beneficial role on cognitive functions by acting on different regions of the brain involved in cognitive and executive tasks such as the prefrontal cortex and hippocampus. All conditions that cause a loss of estrogen levels, such as the menopause, can anticipate or exacerbate the normal decline in cognitive function induced by aging [12]. Moreover, women with early menarche have estradiol (E2) levels approximately two to three times higher than women with menarche at age 13 or older [13]. There is extensive evidence in the literature that E2 is a neurotrophic factor that regulates the development of numerous areas of the brain involved in cognitive functioning, including the striatum, hippocampus, and cerebral cortex [14] by sustaining neurogenesis and enhancing synaptic transmission [15]. We found that a later age at menarche, considered a possible factor delaying estrogen stimulation of the brain areas, was associated with more severe cognitive decline in our patients with PD. In support of our result, other authors have shown that an age at menarche ≥16 years is associated with a 23% higher risk of developing dementia compared to menarche at age 13 in women from the general population [16]. This study showed that also earlier menopause is associated with elevated risk of dementia. It is interesting that in our cohort ages at menarche but not age at menopause or duration of fertility correlate with MoCA scores. Menarche is a transition event characterized by a great increase in levels of estrogen. We speculate that this massive exposure to estrogen is important for enhancing neuroplasticity and for conferring a future protective role to the brain. Moreover, an early menarche and consequently an early onset of estrogen action during brain development could improve and drive the plasticity of the CNS, while a late menarche could reduce hormonal-related changes in the brain, contributing to a lower estrogen-mediated modeling. It has been shown that earlier puberty is associated with a more rapid maturation of subcortical and frontal regions in females [17], or that circulating estrogen levels during sexual maturation are associated with a larger parahippocampal volume in the developing brain of females [18]. All the above-mentioned areas are involved in multiple cognitive tasks, including verbal or working memory and executive functions. Since these regions of the brain are estrogen sensitive, their lesser structuring by estrogens during fertile life due to a late menarche can compromise some cognitive abilities. According to our hypothesis, the brain of women at an earlier age at menarche are more susceptible to estrogen-mediated neuronal plasticity phenomena than women with later menarche. Lack of association between age at menopause or duration of fertile life might argue for greater neuroprotection mediated by timing of menarche in comparison to timing of menopause. In particular, timing of cessation of estrogen exposure or its duration are less important for ensuring neuroprotection from CD. Differently, the initiation of estrogen exposure had more definitive and significant effects on the risk of CD. In a recent study, it has been revealed that late menarche and not shorter reproductive period might be associated with lower performances on cognition as assessed by mini-mental state examination [19]. A possible explanation of this finding, as already proposed by the group of Chou MT [19], is that some cognitive functions, especially those regulated by the prefrontal cortex, such as executive function, learning, and planning, rise to their developmental state about the age of 12 years [20]. In our study, this was the median value of people with MoCA equal or higher than 26. The presence of an age-sensitive menarche window has been proposed in a recent investigation on 1082 women in which menarche at the age between 11 and 14 years has been associated with better cognitive performance in comparison to women with menarche at the age of 15–17 years old [21].

### 4.2. Estro-Progestinic Therapy

No statistical significance was obtained when we assessed the contribution of EP therapy during fertile life to improve cognition. In the literature, few reports are available about the role of exogenous pre-menopausal estrogens in PD, and contrasting findings can be found in [22]. The use of pre-menopausal EP therapy seemed to enhance some cognitive abilities such as visuo-spatial ability and speed and flexibility in healthy cognitive people [23]. However, based to the best of our knowledge, no data deeply explored the role of pre-menopausal estrogens or EP therapy in cognition of women with PD.

### 4.3. Pregnancy

Pregnancy, like menarche, is a period of women’s life characterized by changes in estrogen exposure [24]. During pregnancy, several changes in hormonal concentrations occur. Women during pregnancy, in fact, are exposed to an increase in hormonal secretion. In particular, progesterone, estradiol, and glucocorticoid levels tend to increase with each trimester of pregnancy, reaching a peak during the third trimester [25]. These changes are needed to adapt the body for new physiological demands, but they also act as drivers of brain changes, modulating the plasticity of various neuronal populations, including hippocampal neurons. Most studies exploring the cognitive performance of pregnant women show that during pregnancy, and especially during the late phase, there is a decline in some cognitive functions, especially in memory [26]. This clinical observation is confirmed by some animal studies. For example, hippocampal volume was also reduced in pregnant rats with impaired spatial memory. However, long-term evaluations have shown the same pregnant rats scored better in spatial learning and memory than age-matched non-pregnant rats [27], suggesting that pregnancy might ensure long-term beneficial changes in cognitive function and may explain the better results obtained by the group of our patients with longer cumulative periods of pregnancies. We found no influence of pregnancy and number of pregnancies on patients’ cognitive outcomes. This seems to suggest that, although a massive exposure to estrogen occurs both during pregnancy and during menarche, the effects of estrogen stimulation during menarche exert maximal protection for cognitive health.

### 4.4. Hormone Replacement Therapy

Some authors have suggested that the post-menopausal administration of estrogens seemed to mitigate motor fluctuations in some cases [28] and improve motor symptoms especially in patients with early PD onset [29], but opposite findings can be outlined from the literature [30]. A protective effect of HRT is described for cognitive decline in normal subjects [31]. Moreover, a previous study found that post-menopausal HRT reduces risk for developing dementia in people with PD [32], and another that HRT can reduce the risk of developing PD [33], despite not supported by subsequent meta-analyses [34]. Despite these results, our study showed that the use of HRT during post-menopause did not reduce cognitive or motor complaints in women with PD. This can be related to our limited sample. A more extensive investigation, especially with a case–control design and on a larger number of patients, is necessary. Another finding may relate to the loss of the therapeutic window of HRT, which has been hypnotized to be very restricted and, therefore, may not allow some patients to benefit on cognitive functions [35]. However, considerations coming from investigations into other neurological conditions with cognitive decline, in particular AD, recapitulate the same opposite findings outlined in the case of PD. In fact, if some studies showed a protective effect of estrogen in reducing the risk of dementia, other works did not confirm this [36]. An issue that is advocated from studies on AD is also to appropriately assess the effect of the timing of the HRT administration [36]. Also in our study, which was an exploratory one, this issue was not explored.

## 5. Conclusions

Our study suggests a protective role on cognitive decline in women with PD given by early age at menarche in women with PD. Inversely, an increased risk of late age at menarche can be speculated. No association was found between cognitive decline and age at menopause or fertile life duration. This suggests that the timing of initiation of estrogen stimulation is more important than its end or its duration. However, although the preclinical data suggest a major role of estrogens in cognition, translational knowledge in the human brain is lacking. A possible explanation of the influence of estrogens levels on cognition may lie in the distribution of estrogen receptors (ERs) throughout the brain. ERs are in fact located in areas mainly involved in learning and memory, such as the prefrontal cortex, dorsal striatum, nucleus accumbens, amygdala and, above all, hippocampus [37]. In particular, estrogens seemed to be essential for the development of the gyrus dentate, in which a great distribution of their receptors has been found [37]. Neurogenesis of the hippocampus, especially of the dentate gyrus, has been also highlighted in adults, as shown in animal models and more recently also in the human brain [38]. We speculate that the great peak of estrogen during the menarche might stimulate these receptors and enhance neurogenesis in the dentate gyrus of the hippocampus, conferring a future protective role in cognition. It is known that the capacity of neurogenesis diminishes during aging. This could explain why earlier age at menarche may ensure neuroprotection in comparison to the other reproductive lifespan features. In this way, cognitive abilities could benefit from the phenomena of synaptic plasticity. However, no experimental demonstration of this hypothesis is currently available, or this mechanism has never been investigated. Moreover, this hypothesis needs to be tested also in people who are not affected with PD. Another neurodevelopmental explanation could be found in the coincidence of the peak in estrogens during an early menarche and the maximum degree of maturation of the prefrontal cortex [39]. In conclusion, our observation remains preliminary, and a higher number of people is necessary to confirm the results of our study, as well as the presence of a control group with healthy people. A prospective longitudinal design can better help to explore the real contribution of each single reproductive lifespan variable. Moreover, an inclusion of the comorbidities and assessments for anxiety and depression—not conducted in our study—may concretely assess the role of likely confounding factors.

## Figures and Tables

**Table 1:** Clinical, demographic, and reproductive features of patients with PD.

	Total (*n* = 90)
Duration of disease (years), median (IQR)	9 (6–13)
Age at onset (years), median (IQR)	63 (56–68)
H&Y (scores), median (IQR)	2 (1–3)
LEDD (dosage in mg), median (IQR)	600 (339–877)
UPDRS (scores), median (IQR)	43.5 (27–63)
MoCA, median (IQR)	23 (17–26)
Age at menarche (years), median (IQR)	12 (12–14)
Age at menopause (years), median (IQR)	50 (48–52)
Natural menopause (%)	85.6 (*n* = 77)
Surgical menopause (%)	14.4 (*n* = 13)
Abortion (%)	45.6 (*n* = 41)
Numbers of abortion, median (IQR)	1 (1–2)
Fertility time (years), median (IQR)	38 (35–40)
Delivery (%)	91.1 (*n* = 82)
No. of delivery (n)	2 (1–3)
Breast feeding (%)	45.6 (*n* = 41)
Total time of breastfeeding (months), median (IQR)	5 (3–9)
CLP (months), median (IQR)	18 (10–27)
EP therapy (%)	27.8 (*n* = 25)
EP duration (months), median (IQR)	60 (12–96)
Age at EP starting (years), median (IQR)	25 (22–31)
HRT (%)	16.7 (*n* = 15)
HRP duration (months), median (IQR)	24 (10–28)
Age at HRP starting (years), median (IQR)	48 (45–52)

MoCA: Montreal Cognitive Assessment scale. H&Y: Hoen and Yahr. LEDD: levodopa equivalent dose. UPDRS: Unified Parkinson’s Disease Rating Scale. CLP: Cumulative Length of Pregnancy. EP: Estroprogestinic. HRT: Hormone Replacement Therapy. Data are expressed as median with interquartile range (IQR) or percentage, as appropriate.

**Table 2:** Differences in clinical, demographic, and reproductive features of patients with PD in relation to MoCA scores.

	MoCA < 26 (*n* = 63)	MoCA ≥ 26 (*n* = 27)	*p* Value
Duration of disease (years), median (IQR)	9.4 (6–12.7)	8.3 (6.1–12.7)	0.9
Age at onset (years), median (IQR)	65 (59.5–68)	58 (50–63.5)	0.001
H&Y (scores), median (IQR)	2 (1–3)	1 (1–2)	0.02
LEDD (dosage in mg), median (IQR)	650 (327.5–1010)	460 (356–723)	0.2
UPDRS (scores), median (IQR)	46 (33.5–64.5)	36 (21.5–46.5)	0.002
Age at menarche (years), median (IQR)	13 (12–14)	12 (11–12)	<0.0001
Age at menopause (years), median (IQR)	50 (48–52)	50 (47.5–54)	0.5
Natural menopause (%)	85.7 (*n* = 54)	87.5 (*n* = 23)	0.6
Surgical menopause (%)	14.3 (*n* = 9)	14.8 (*n* = 4)	
Abortion (%)	42.9 (*n* = 27)	51.9 (*n* = 14)	0.3
Numbers of abortion, median (IQR)	1 (1–2)	1 (1–1)	0.2
Fertility time (years), median (IQR)	37 (35–39)	39 (36.5–42)	0.01
Delivery (%)	90.5 (*n* = 57)	92.6 (*n* = 25)	0.5
No. of delivery (n)	2 (1–3)	2 (1.5–3)	0.5
Breast feeding (%)	42.9 (*n* = 27)	51.9 (*n* = 14)	0.3
Total time of breastfeeding (months), median (IQR)	6 (4–9)	4 (3–5)	0.1
CLP (months), median (IQR)	18 (10–26.5)	18 (12.5–27)	0.8
EP therapy (%)	13.3 (*n* = 12)	14.4 (*n* = 13)	0.004
EP duration (months), median (IQR)	60 (5.5–150)	60 (24–60)	0.8
Age at EP starting (years), median (IQR)	24.5 (21.5–25)	27 (24–35)	0.1
HRT (%)	17.4 (*n* = 11)	14.8 (*n* = 4)	0.5
HRP duration (months), median (IQR)	24 (10–29)	21 (12–26)	0.7
Age at HRP starting (years), median (IQR)	48 (46–51)	47.5 (45–52.5)	0.9

MoCA: Montreal Cognitive Assessment scale, adjusted for age and education: H&Y: Hoen and Yahr. LEDD: levodopa equivalent dose. UPDRS: Unified Parkinson’s Disease Rating Scale. CLP: Cumulative Length of Pregnancy. EP: Estroprogestinic. HRT: Hormone Replacement Therapy. Data are expressed as median with interquartile range (IQR) or percentage, as appropriate.

**Table 3:** Univariate and multivariate analysis exploring the effects of variables linked to fertility on MoCA.

Predictors	Univariate				Multivariate			
	B	OR	95% CI	*p*	B	aOR	95% CI	*p*
Duration of disease	0.01	1.01	0.9–1.1	0.7				
Age at evaluation	−0.07	0.9	0.8–0.9	**0.003**	−003	0.9	0.9–1.04	0.4
LEDD	−0.001	0.9	0.9–1	0.1				
HY	−0.69	0.5	0.2–0.8	**0.02**	−0.3	0.7	0.3–1.5	0.4
UPDRS	−0.041	0.96	0.93–0.98	**0.003**	−0.021	0.9	0.9–1.01	0.2
Age at menarche	−0.7	0.49	0.3–0.7	**<0.0001**	−0.6	0.5	0.3–0.8	**0.005**
Age at menopause	0.05	1.05	0.9–1.2	0.4				
Natural menopause	−0.04	0.9	0.3–3.4	0.9				
LGC	0.003	1	0.9–1.04	0.8				
Breast feeding	0.3	1.4	0.5–3.5	0.4				
Cumulative BF	−0.08	0.9	0.8–1.03	0.6				
Delivery	0.2	1.3	0.2–6.9	0.7				
Number of deliveries	0.01	1.01	0.7–1.5	0.9				
Abortion	0.3	1.4	0.5–3.5	0.4				
No. of abortion	−0.79	0.45	0.1–1.4	0.1				
EP therapy	1.3	3.9	1.4–10.5	**0.006**	0.7	2.1	0.6–6.9	0.1
Duration of EP therapy	−0.009	0.9	0.9–1	0.17				
Age at EP therapy	0.17	1.2	0.9–1.4	0.07				
HRT								
Duration of HRT	−0.02	0.9	0.9–1.06	0.6				
Age at HRT	0.006	1	0.7–1.3	0.9				

Bold font indicates statistical significance (*p* < 0.005).

## Data Availability

Data are available from the corresponding author upon reasonable request.
